# A Fast Level Set Method for Synthetic Aperture Radar Ocean Image Segmentation

**DOI:** 10.3390/s90200814

**Published:** 2009-02-03

**Authors:** Xiaoxia Huang, Bo Huang, Hongga Li

**Affiliations:** 1 Institute of Remote Sensing Applications, Chinese Academy of Sciences, Beijing 100101, China; Tel: (86)-10-64850282; E-mails: hxx@irsa.ac.cn, E-mail: lihongga@irsa.ac.cn; 2 Department of Geography and Resource Management, The Chinese University of Hong Kong, Shatin, NT, Hong Kong

**Keywords:** Image Segmentation, Fast Level set, Synthetic Aperture Radar Ocean Image

## Abstract

Segmentation of high noise imagery like Synthetic Aperture Radar (SAR) images is still one of the most challenging tasks in image processing. While level set, a novel approach based on the analysis of the motion of an interface, can be used to address this challenge, the cell-based iterations may make the process of image segmentation remarkably slow, especially for large-size images. For this reason fast level set algorithms such as narrow band and fast marching have been attempted. Built upon these, this paper presents an improved fast level set method for SAR ocean image segmentation. This competent method is dependent on both the intensity driven speed and curvature flow that result in a stable and smooth boundary. Notably, it is optimized to track moving interfaces for keeping up with the point-wise boundary propagation using a single list and a method of fast up-wind scheme iteration. The list facilitates efficient insertion and deletion of pixels on the propagation front. Meanwhile, the local up-wind scheme is used to update the motion of the curvature front instead of solving partial differential equations. Experiments have been carried out on extraction of surface slick features from ERS-2 SAR images to substantiate the efficacy of the proposed fast level set method.

## 1. Introduction

From the time of its inception, Synthetic Aperture Radar (SAR) imaging systems have provided a remote sensing resource complementary to optical and thermal-infrared sensors. SAR Imagery has been applied in a gamut of areas including ecological observation, surface monitoring, target detection, and mapping. Primary among the advantages of SAR imaging are day-night and all-weather operation, wide area coverage and sensor height-independent image resolution. These functionalities have also led to an escalating interest in the last decade towards the usage of SAR imagery for ocean environment monitoring.

Nevertheless, the multiplicative speckle noise caused by microwave illumination degrades the quality of the SAR imagery. Speckle impedes visual interpretation of SAR images and may lead to interclass confusion. This is particularly so in the quick detection of oil slicks on the sea surface. The low backscatter cross section of the surface of oil spillages causes Bragg wave dampening effects on the sea. As a result of this an oil spill comes out on the image as a dark slick or dark spot, whereas the surrounding water appears bright. Consequently, oil slicks in SAR images are characterized by a high noise and low contrast, disturbing their extraction and analysis.

A variety of methods have been developed to reduce speckles. For example, geometric filters such as those of Frost [1] and Lee [2] have been designed. However, convolving the image with filters could reduce the segmentation accuracy by blurring the feature boundaries due to high noise [3]. Traditional edge segmentation methods, such as border tracing, have drawbacks as well. In these methods, use of a small convolution window to preserve image resolution generates an edge map with incomplete feature boundaries and spurious edge points, due to high noise. In recent years, several novel methods have been attempted for reducing speckles while preserving edges such as Markov Random Fields [4][5] and wavelet methods [6]. Unlike these methods, level set provides a new option that is able to remove high noise and simultaneously delineate a smooth and stable boundary [7].

Level set was proposed by Osher and Sethian [8] as an interface propagation technique for various applications including image segmentation. In this method, a curve is embedded as a zero level set of a higher dimensional surface. Following that, the entire surface is evolved to minimize a metric defined by the curvature and image gradient, i.e. the speed terms of the level set will reduce to zero when reaching the object boundary. The level set interface can propagate with topological changes, significant protrusion and narrow regions.

In the traditional level set technique, instead of tracking the points on the interface itself, the interface is embedded as the zero level set propagates by iterations. For this reason the standard level set algorithm could possibly be rather slow for real-time or near real-time image processing. Algorithms such as the narrow-band algorithm [9], fast marching algorithm [10] and the two-cycle fast algorithm [11] have been developed to make this process more efficient.

Based upon our previous work using a level set for image segmentation [7], this paper proposes a more efficient, three-component based level set algorithm for noisy SAR image segmentation. Firstly, it operates in a grid domain, which updates the adjoining pixels around the zero level set, and only the boundary and neighboring pixels in the propagation direction are considered in the computation process. Next, a single list is selected to track the interface's properties by keeping pace with the propagation boundary to shorten time complexity. At last, the complicated partial differential equations are simplified into a local up-wind scheme to reduce numerical computation time. Moreover, an intensity model and a curvature model are applied for noise removal and simultaneous trouble-free extraction of surface slicks. To illustrate the effectiveness of the proposed method, experiments were conducted on extracting surface slick features from ERS-2 SAR ocean images. In addition the proposed techniques were evaluated against other level set methods such as fast marching, and the results confirmed the efficiency gains of the proposed method.

## 2. Background of Level Set

### 2.1 Level set method

Level set is an efficient numerical technique for interface propagation. A brief introduction of this method is given here. The detailed explanation can be found in Sethian (1999)[10].

**Figure 1.**
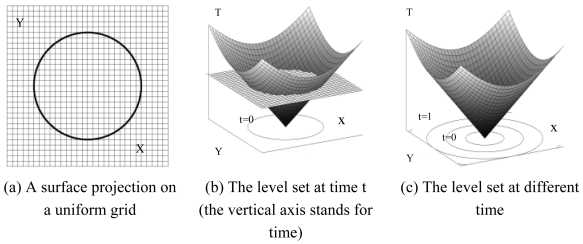
Illustration of level sets.

In the level set method, a scalar Lipschitz function, *ϕ*(x, t) (also known as level set function), defines the embedding of an n-dimensional surface in an R^n+1^ space surface, where x ∈ R^n+1^, and t = time t (Figure 1). The set of points on the surface, S, are mapped by *ϕ* such that:

(1) S={x∣ϕ(x,t)=k}

where k is an arbitrary scalar value, namely S is the k level set of *ϕ*.

The essential idea of the level set is to represent the moving front ∂*ϕ* as the zero level set of the time-dependent level set function *ϕ* :{*ϕ*(x, t = 0) = 0}.

Moreover, the level set is supposed to be topology free, since different topologies of the zero level set do not imply the different topologies of a level set *ϕ*.

In accordance with the propagation of the front, the first order partial differential equation (PDE) of the level set is represented as:

(2) $$\frac{\partial \phi }{\partial t}=F\left|\nabla \phi \right|$$

where *F* is a scalar function that defines the speed in the outward direction normal to *ϕ*, |∇*ϕ*| represents some appropriate finite different operators for the spatial derivative, and ∂*t* the time step.

The speed term in which the front propagates is defined by the function *F*:

(3) $$F=F_{\text{prop}}+F_{\text{curv}}+F_{\text{adv}}$$

where *F_prop_* is the propagation expansion speed, *F_curv_* = −∈*κ* is the dependence of speed on the curvature, and *F_adv_* is the advection speed. Note that ∈ denotes some constants greater than zero and k is a curvature of a planar curve. While dealing with 2D images, the advection factor *F_adv_* is not considered as part of the front motion. Hence, the two main speed terms, i.e., *F_prop_* and *F_curv_*, are employed to deal with SAR images, where *F_prop_* is derived from the image intensity gradient, and *F_curv_* from the curvature flow.

### 2.2 Fast level set methods

As the level set method is formulated from numerical equations for interface propagation, the iteration periods of the standard algorithm for boundary expansion are invariably longer. Taking into consideration a single pixel and its neighboring pixels, one solution is obtained by updating the value of each pixel till the final boundary is reached. For such a solution, O(*N^2^*) operations per time step are needed. Assuming the total number of iterations to be N, no less than O(*N^3^*) iterations will be needed. To overcome the problem of longer time requirement, fast level set methods such as narrow-band level set and fast marching method have been introduced.

#### (1) Narrow-band level set

**Figure 2.**
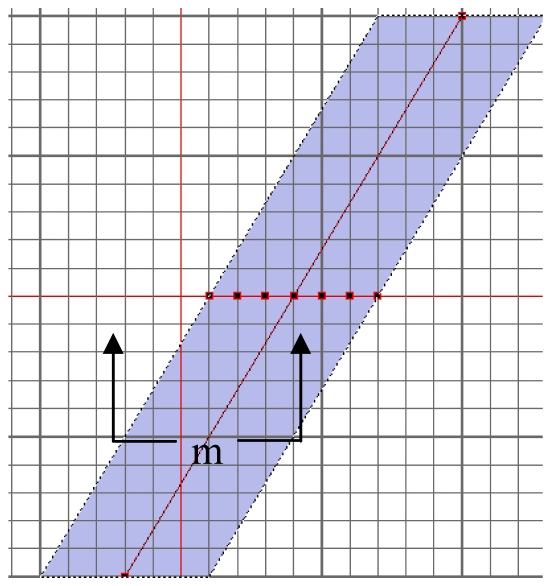
Narrow-band of level set.

Level set computations are usually carried out using the narrow-band algorithm as described by Malladi *et al.* [8]. The narrow-band algorithm, however, limits the propagation front's requirements to update the properties of the neighboring pixels around the zero level set. As shown in Figure 2, the entire two-dimensional grid of data is stored in a square array.

A one-dimensional array is employed to keep track of the points in the narrow band. Assuming the number of points in the front to be *k*, the band width to be *m*, the number of iterations to be *N*, the operation count drops down to O(*kmN*). In the worst possible situation, the narrow band method will at most reduce the total operation count to (*N^3^*). Even though this indicates a significant progress over the brute-force approach, it is still considered slow for (near) real-time image processing applications [9].

#### (2) Fast marching level set

In a situation wherein the speed function depends only on the interface position, the speed function *F* (Equation 7) will be reduced to *F* = *F_curv_*. Furthermore, if *F_curv_* > 0, it would be sufficient to solve the stationary perspective boundary problem |∇*T*|*F* = 1, given that *x:T(x)* = *0* (where T is the time of arrival of the interface at a particular point). The level set in the aforementioned situation can be efficiently solved by utilizing the fast marching algorithm developed by Sethian [9]. This algorithm marches downwind from the known value and computes new values at each of neighboring grid points. Furthermore, it also uses a min-heap data structure to store boundary value points, which enables solving the boundary value without iteration.

A min-heap is a complete binary tree in which the value at any given node is less than or equal to the value of its child nodes. In general, finding the smallest element in the min-heap requires O(*1*) time and the remaining elements (say M in number) satisfying the heap property, such as ‘insert’ and ‘delete’ consume O(*log M*) time. Hence in the worst scenario, for the fast marching method, the total heap operations that include an insert element, a ‘find’ and ‘delete’ smallest element will be O(*2MlogM*) on a grid with M points. In case of a two-dimensional grid with *N* points in each direction, the fast marching method reduces the total operation count to (*4N^2^logN*).

## 3. The proposed fast level set method

On the whole, the fast marching method is more efficient than the narrow-band algorithm; however, it does not take into account the effect of image intensity, which attracts the interface inward or outward. Moreover, on above methods, the PDE is solved with a tube in the neighborhood of the zero level set, and at every iteration the level set function should be reinitialized and pixels in the tube be updated dynamically by solving a PDE for a fixed number of steps. This might well result in a tendency to ‘leakage’. Thus, in spite of the advances made by the aforementioned level set methods, there still exists room for more improvement.

In image processing, the accuracy is usually limited by pixel size, especially for the segmentation in a noisy image, the achieved boundary is expected to be smooth and quick. The algorithm proposed herein, in addition to incorporating the advantages of the aforementioned methods, also functions more efficiently. Firstly, considering high noise in the SAR ocean image, a level set formulation is ingrained with the intensity and curvature models to construct extension velocity, in order to handle topological changes automatically. Subsequently, refer to narrow-band and fast marching methods, the tube is simplified to four neighborhood pixels just near the zero level set, and a list is used to replace min-heap in order to accelerate. Finally, the upwind partial differential equation approximation is used to optimize reinitialization and propagate the boundary with available information to a stable boundary.

### 3.1 Intensity model

In a SAR image, the intensity difference is regarded as the primary factor influencing the front propagation of an oil slick edge. Therefore, *F_prop_* is derived from the intensity distribution of the pixels:

(4) $$\begin{array}{c} I_{\text{average}}=(I_{\text{lower}}+I_{\text{high}})/2\\ F_{\text{prop}}=\{\begin{array}{lll} I(x,y)-I_{\text{lower}} & \text{if} & I(x,y)<=I_{\text{average}}\\ I_{\text{high}}-I(x,y) & \text{if} & I(x,y)>=I_{\text{average}} \end{array} \end{array}$$

where *I(x, y)* denotes the intensity of a pixel at the position of x, y, while *I_lower_* and *I_high_* denote the minimum and maximum thresholds of an oil slick, respectively. This intensity model allows the level set surface to move towards the image pixels with grayscale values between *I_lower_* and *I_high_*.

### 3.2. Curvature model

Besides intensity, curvature is another important factor in determining the propagation. The mean curvature of the front at any point can be obtained from the divergence of the unit normal vector to the front, i.e.:

(5) $$\kappa =\nabla •\frac{\nabla \phi }{\left|\nabla \phi \right|}=\frac{\phi_{xy}\phi_{y}^{2}-2\phi_{y}\phi_{x}\phi_{xy}+\phi_{yy}\phi_{x}^{2}}{{\left(\phi_{x}^{2}+\phi_{y}^{2}\right)}^{3/2}}$$

An up-wind partial differential scheme is employed to obtain the stability of a boundary. This scheme is based on a one-sided derivative that looks in the up-wind direction of the moving wave front, thereby avoiding the over-shooting associated with finite forward differences.

To compute the derivatives for the level set, three difference operators, i.e. the forward difference D^+^, backward difference D^-^, and central difference, are used with the eight neighboring pixels. For instance, the differences in the x direction on the SAR image with spacing h at time t, i.e. u(x, y, t), are defined by first and second order terms as:

(6) $$\begin{array}{l} \{\begin{array}{c} D_{x}^{+}u(x,y,z)=\frac{u(x+h,y,t)-u(x,y,t)}{h}\\ D_{x}^{-}u(x,y,z)=\frac{u(x,y,t)-u(x-h,y,t)}{h}\\ D_{x}u(x,y,z)=\frac{u(x+h,y,t)-u(x-h,y,t)}{2h} \end{array}\\ \{\begin{array}{c} D_{x}^{+y}u(x,y,t)=\frac{u(x+h,y+h,t)-u(x-h,y+h,t)}{2h}\\ D_{x}^{-y}u(x,y,t)=\frac{u(x+h,y-h,t)-u(x-h,y-h,t)}{2h}\\ D_{y}^{+x}u(x,y,t)=\frac{u(x+h,y+h,t)-u(x+h,y-h,t)}{2h}\\ D_{y}^{-x}u(x,y,t)=\frac{u(x-h,y+h,t)-u(x+h,y-h,t)}{2h} \end{array} \end{array}$$

where $D_{x}^{+}$ computes the new value at (x, y) and (x+h, y), hence information for the solution propagates from right to left. $D_{x}^{-}$ computes the new value at (x, y) and (x-h, y), thus information for the solution propagates from left to right. *D_x_* computes the new value at (x+h, y) and (x-h, y), thus information for the solution propagates from both sides.

In a similar manner, the forward, backward or centered Taylor series expansions in the x direction can be derived:

(7) $$\{\begin{array}{ccc} \text{Forward} & \text{scheme} & u(x+h,y,t)=u(x,y,t)-hD^{+x}u(x,y,t)\\ \text{Backward} & \text{scheme} & u(x+h,y,t)=u(x,y,t)-hD^{-x}u(x,y,t)\\ \text{Centered} & \text{scheme} & u(x+h,y,t)=u(x-h,y,t)-2hD^{x}u(x,y,t) \end{array}$$

The curvature is computed using the above derivatives and the difference of the normals method introduced by Whitaker and Xue (2001)[12]. The two normals, n^+^ and n^-^, are then computed by:

(8) $$\begin{array}{l} n^{+}=\left\{\begin{array}{c} \frac{D_{x}^{+}}{4\sqrt{{\left(D_{x}^{+}\right)}^{2}+{\left(\frac{D_{y}^{+x}+D_{y}}{2}\right)}^{2}}}\\ \frac{D_{y}^{+}}{4\sqrt{{\left(D_{y}^{+}\right)}^{2}+{\left(\frac{D_{x}^{+y}+D_{x}}{2}\right)}^{2}}} \end{array}\right\}\\ n^{-}=\left\{\begin{array}{c} \frac{D_{x}^{-}}{4\sqrt{{\left(D_{x}^{-}\right)}^{2}+{\left(\frac{D_{y}^{-x}+D_{y}}{2}\right)}^{2}}}\\ \frac{D_{y}^{-}}{4\sqrt{{\left(D_{y}^{-}\right)}^{2}+{\left(\frac{D_{x}^{-y}+D_{x}}{2}\right)}^{2}}} \end{array}\right\} \end{array}$$

The components of divergence are then computed as:

(9) $$\left\{\begin{array}{c} \frac{\partial n_{x}}{\partial x}=n_{x}^{+}-n_{x}^{-}\\ \frac{\partial n_{y}}{\partial y}=n_{y}^{+}-n_{y}^{-} \end{array}\right\}$$

Finally, the curvature model is formulated as:

(10) $$F_{\text{curv}}=\frac{\partial n_{x}}{\partial x}+\frac{\partial n_{y}}{\partial y}$$

Using this equation, the direction and velocity of the speed dependent on the curvature can be easily derived. The propagating front of an initial level set to the object boundary starts from inside the selected region, and grows outward. This outward growth is influenced by the image intensity and curvature. The front propagates till the speed function *F* reduces to near zero and finally stops at the shape boundary. Accordingly, the final algorithm works as follows.

(11) $$\phi_{ij}^{n+1}=\phi_{ij}^{n}+\Delta t(F_{\text{prop}}|\nabla_{ij}\phi_{ij}^{n}|-\in \kappa |\nabla_{ij}\phi_{ij}^{n}|)$$

The initial estimation of *ϕ* is propagated forward in time via the up-wind scheme. To assure a stable solution, the up-wind scheme approximates *ϕ* using one-sided derivatives that are always in the up-wind direction of the propagating surface.

### 3.3 Fast Algorithm Implementation Flowchart

The implementation of the proposed level set method has been done through the extraction of the surface slick boundaries. For improving computation efficiency, a list is used to record the inside or upwind side neighboring pixels, and a 2-dimenation array for storing the level set function *ϕ*. We define *ϕ* as follows:

(12) $$\phi (\text{x})=\{\begin{array}{ll} 1 & \text{if x is exterior or downwind pixels}\\ -1 & \text{if x is inside or upwind neighbor pixels}\\ -2 & \text{if x is interior or upwind pixels} \end{array}$$

**Figure 3.**
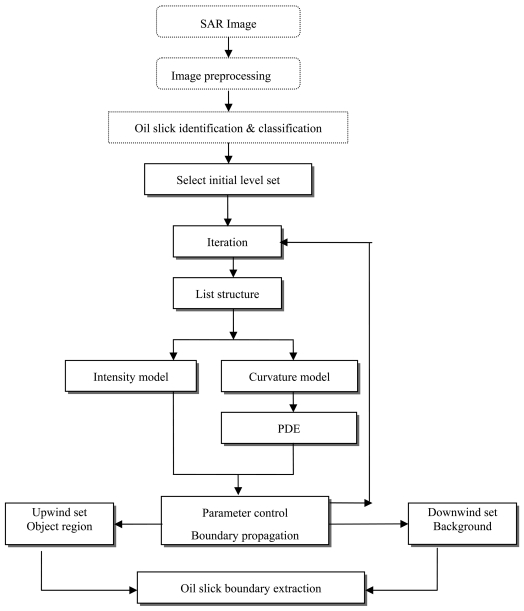
SAR image segmentation using the proposed fast level set method

As shown in Figure 3, the procedural implementation flowchart is as follows:

1. A single seed (or multiple seeds) of surface slick signatures on the image is (or are) selected and used as the starting interface (i.e. the zero level sets). The grayscale upper and lower limits (i.e. *I_lower_* and *I_high_*) are selected according to the surface slick characteristics in the SAR imagery (i.e. the grayscale value of the pixels inside and around the boundaries of the surface slicks).
2. The pixels in the image are divided into two sets: the upwind set wherein the pixels are in the interface with *ϕ* equal to -1 or -2, and the downwind set in which the pixels are away from the interface with *ϕ* equal to 1. The pixels in the list record upwind neighbor pixels near to propagation curvature and represent the uncertain boundary.
3. Subsequently, the front pixel in the list is popped and tagged as the upwind known set. Four neighboring pixels are selected and their speed functions F are calculated. Then, update $\phi_{ij}^{n+1}$ by using the intensity model and the curvature model based on partial differential equation.
4. Fast update level set function. If a pixel is located upwind neighboring to curvature and $\phi_{ij}^{n+1}$ is less than zero, update its $\phi_{ij}^{n+1}$ to -2; and $\phi_{ij}^{n+1}$ is more than zero, update its $\phi_{ij}^{n+1}$ to 1. On the contrary, if a pixel is located downwind neighboring to curvature and $\phi_{ij}^{n+1}$ is less than zero, update its $\phi_{ij}^{n+1}$ to -1 and push the pixel into list. Above first outward then inward updating process limits level set distance function into a narrow band by, and simply the re-initialization calculation.
5. Lastly, all pixels in the list will be subjected to computation until none of it remains in the list.

**Figure 4.**
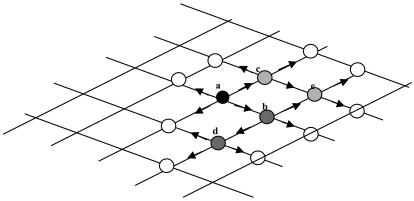
Example of processing stages by the proposed fast level set method.

The above algorithm is further illustrated by an example in Figure 4. Pixel *a* is assumed to be a seed and the object region and background are divided into two sets. In order to improve the object boundary propagation efficiency, a list is adopted, which aids tracing the boundary change. List serial operations such as pop_front, push_back and deletion pixel during each iteration are employed to enhance performance. Pixel *a* is set with an initial value and pushed back into the list. Then, front pixel *a* is popped to compute the value of its neighboring pixels by using the intensity model and the curvature model. The use of the up-wind PDE is applied to calculate downwind and upwind neighboring pixels level set value. Furthermore, if the $\phi_{ij}^{n+1}$ values of downwind neighboring pixels such as *b* and *c* less than zero, then curvature move outwards, thus updating level set distance function and leading to push *b* and *c* into the back of list. This completes the first iteration of boundary propagation. The iterations are carried on end till no pixel is left in the list.

The stable segmentation boundary, which distinguishes the surface slick from different background such as sea surface and land, serves as the line between the upwind set and downwind set. While selecting a known surface slick pixel as an inner seed in the oil slick, only the interface front expanding outwards is considered. Hence if an outer seed (i.e. non surface slick pixel) is selected, the interface front will expand inwards.

The list is a simple but efficient data structure. In the worst scenario, the operation for push back, pop front, inserting, or deleting element take O(*logM*) time assuming there are M elements in the list. As a result of using list to replace min-heap, the efficiency of inserting the downwind side pixel decreases from O*(logM)* to O*(1)* (logM is usually far more than 1). Although the calculation both outward then inward for updating level set distance value maybe consume compute time, but the time complexity of proposed method will decreases from O*(2MlogM) to* O*(2M)* in comparison with traditional fast march method.

## 4. Experimental Results

**Figure 5.**
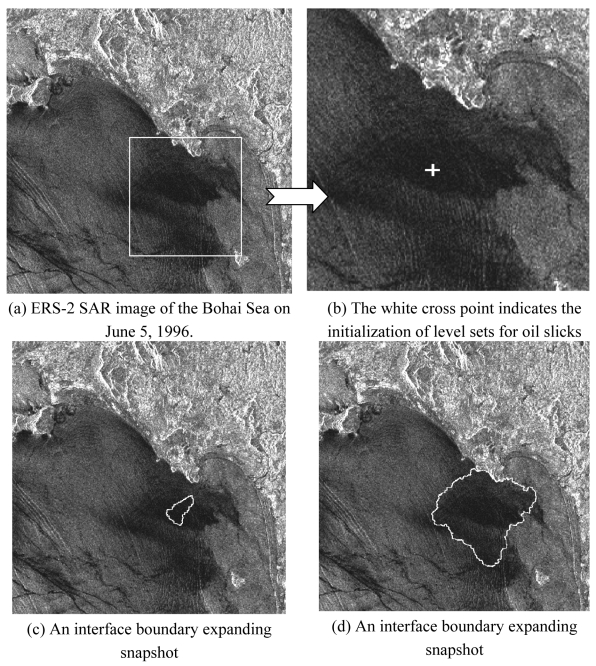

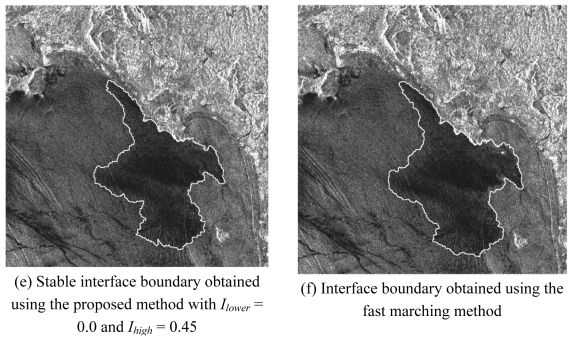
The proposed fast level set method with single seed for initialization of level sets.

**Figure 6.**
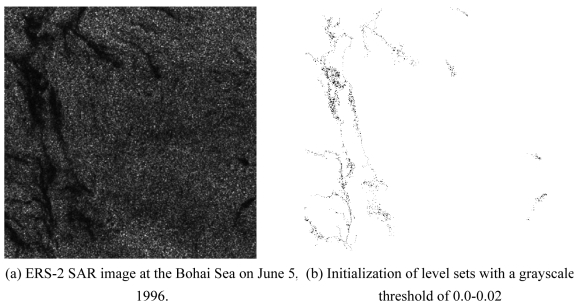

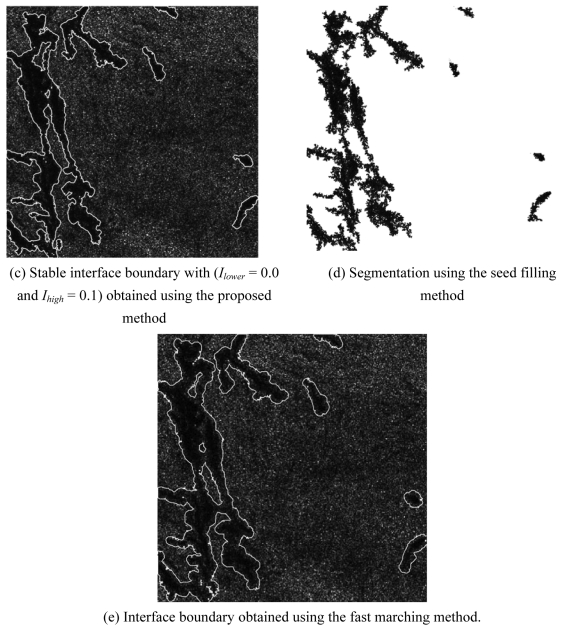
The proposed fast level set method with multiple seeds for initialization of level sets.

To confirm the efficiency gain of the proposed level set method as shown in Table 1, two 1024 × 1024 pixel SAR images (Figures 5 and 6) of the Bohai Sea recorded on June 5, 1996 were used to segment surface slicks with single and multiple initialization of level sets, respectively. In the first experiment, an arbitrary shape, i.e., a point, was manually defined as a single initial level set, whereas in the second experiment, a threshold was set to obtain multiple initial level sets. *I_lower_* and *I_high_* were defined as the lowest and highest grayscale values of the slicks, and were selected according to the grayscale distribution of oil slicks in the radar image. The ratio between the intensity model and the curvature model constrained the smoothness of the evolving surface to prevent some of the leaking that was common in the connected-component structures. If the ratio were equal to 0.0, *i.e.*, only using the curvature model, the proposed method would become the traditional level set segment.

In the first experiment (Figure 5a), the *priori* information, i.e. zero level set was selected as a single seed in the slick at random shown in Figure 5b. In accordance with the procedure described in the previous section, the SAR image was segmented using the proposed level set method. Considering the grayscale distribution of dark slicks in the image (Figure 5a), the lowest and highest grayscales, i.e., *I_lower_* and *I_high_*, were measured and set to 0.0 and 0.45, respectively. In addition, for balancing the intensity and curvature models, the ratio between the former and latter models was adjusted to 50% for *F_prop_* and 50% for *F_curv_*. The sequence of interface propagation is illustrated from Figures 5c to 5e. It can be found that the interface boundary expands from an initial seed point to a stable boundary. The result using the traditional fast marching method, which only depends on the curvature flow, is also shown in Figure 5f. In the boundary with a distinct change such as coast, both methods arrived at a similar shape, but in the region of slicks with a slowly gradual change, the fast marching method obtained a more smooth but enlarged shape.

In the second experiment with a low contrast SAR image (Figure 6a), our program started with a heap filled with multiple seeds, which used the automated thresholding result as the initial level set (Figure 6b). Due to the low grayscale of slicks and low contrast with background, the lowest and highest grayscales, *I_lower_* and *I_high_*, were respectively detected in the image (Figure 6a) and specified as 0.0 and 0.10, respectively. *F_prop_* and *F_curv_* were set as 50% and 50%, respectively. Comparing with the oil slick boundary obtained by the seed filling method with the same seeds (Figure 6d), the boundary extracted by the proposed level set method (Figure 6c) is much smoother.

The result for the fast marching method, which only depends on the curvature flow, is displayed in Figure 6e. It can be observed that the proposed method gave a shape closer to the seed filling result than the fast marching method for an oceanic radar image with high noise and low contrast.

**Figure 7.**
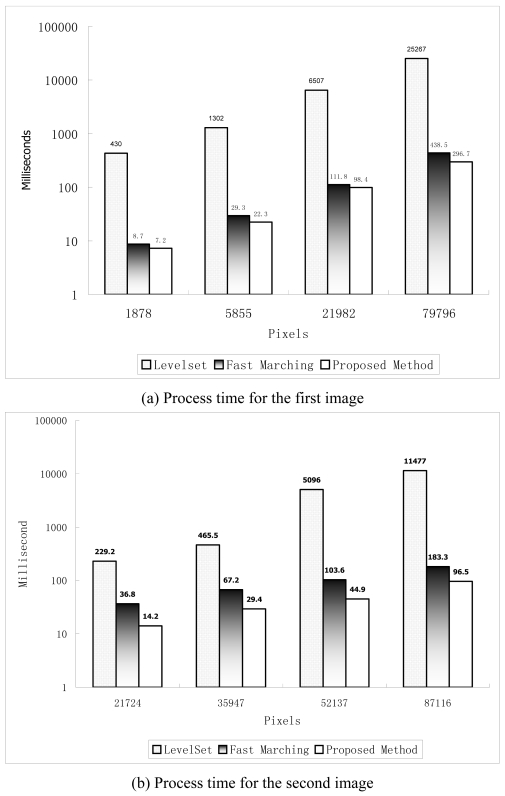
Comparison of the proposed method with the ordinary level set and fast marching methods

To test the efficiency of the proposed method, it was compared with the ordinary level set and fast marching methods in terms of mean processing time for oil slick patches (Figures 7a and 7b). The experiments were performed on a 2.0 GHz Pentium 4 PC, which has 1 Gbytes of memory, 20 Kbytes L1 cache, and 512 Kbytes L2 cache.

As recorded in the calculation, with the first SAR image, the extracted oil slick amounts to 79,796 pixels, whereas in the second image, the extracted oil slick amounts to 87,116 pixels. Apparently, the ordinary level set method lags behind the fast marching and the proposed methods because it has to iterate calculations on the entire image. Although the fast marching and the proposed fast level set methods deal with the pixels close to the slick propagation boundary in a similar way, the proposed method is more efficient than the fast marching method in both the images (Figures 5 and 6) with the single initial seed for the first image (Figure 5) and multiple seeds for the second image (Figure 6). As shown in Figure 7, with the increase of the slick patch size, the efficiency gain tends to be bigger. This indicates that the proposed method is particularly suited for large-size patch processing.

For accuracy assessment, we compared the segmented results using the proposed, seed filling, and fast marching methods with the boundary interpreted manually, and the results for the images in Figures 5 and 6 are listed in Table 1. Clearly, the proposed method achieved a better accuracy than the other two methods.

## 5. Conclusions

In this paper a more efficient level set method for the feature extraction of oil slicks in SAR images has been proposed. It is based on the theory of level set flow propagation, which uses the intensity gradient as the front advancing impetus. The segmentation of oil slicks in oceanic SAR images has been carried out to verify the effectiveness of the proposed method and the results showed that it can arrive at more smooth and ideal boundaries than other level set segmentation methods that only depend on the curvature flow.

The algorithm developed in this paper not only relies on the image intensity and curvature speed functions, but also employs a list to accelerate the interface front processing. The experimental results show that the proposed method is more efficient than other level set methods, especially for large-size image segmentation. This enables the proposed method to be used in near real-time image processing. Furthermore, the high noise can be removed simultaneously and the accuracy of the boundary can be maintained. It is expected that the proposed method epitomizes a prototype for comprehensive processing of high noise and low contrast remote sensing images.

## Figures and Tables

**Table 1.** Accuracy assessment between the proposed, seeding filling, and fast marching methods

		**Proposed method**	**Seed filling**	**Fast marching**

Experiment I (Figure 5)	Area matching error	4.1%	4.9%	9.1%
	Perimeter matching error	22.5%	43.0%	24.7%

Experiment II (Figure 6)	Area matching error	1.9%	3.1%	6.1%
	Perimeter matching error	19.3%	34.3%	23.9%

## References

[1] Frost V.S., Stiles J., Shanmugan K., Holtzmann J. (1982). A model for radar images and its application to adaptive digital filtering of multiplicative noise. IEEE Transactions on Pattern Analysis and Machine Intelligence.

[2] Lee J.S. (1986). Speckle suppression and analysis for synthetic aperture radar images. Optical Engineering.

[3] Durand J.M., Gimonet B.J., Perbos J.R. (1987). SAR data filtering for classification. IEEE Transactions on Geoscience and Remote Sensing.

[4] Dong Y., Forster B.C., Miline A.K. (2003). Comparison of radar image segmentation by Gaussian- and Gamma-Markov random field models. International Journal of Remote Sensing.

[5] Sveinsson J.R., Atli Benediktsson J. (2003). Almost translation invariant wavelet transformations for speckle reduction of SAR images. IEEE Transactions on Geoscience and Remote Sensing.

[6] Achim A., Tsakalides P., Bezerianos A. (2003). SAR image denoising via Bayesian wavelet shrinkage based on heavy-tailed modeling. IEEE Transactions on Geoscience and Remote Sensing.

[7] Huang B., Li H.G., Huang X. (2005). A level set approach to segmentation of oil slicks in SAR images. International Journal of Remote Sensing.

[8] Osher S., Sethian J. (1988). Fronts propogating with curvature-dependent speed: algorithms based on Hamilton-Jacobi formulations. Journal of Computational Physics.

[9] Malladi R., Sethian J.A., Vemuri B.C. (1995). Shape modeling with front propagation: a level set approach. IEEE Transactions on Pattern Analysis and Machine Intelligence.

[10] Sethian J.A. (1999). Level Set Methods and Fast Marching Methods: Evolving Interfaces. Computational Geometry, Fluid Mechanics, Computer Vision and Materials Science.

[11] Shi Y.G., Clem Karl W. (2005). A fast implementation of the level set method without solving partial differential equations, Technical Report ECE-2005-02.

[12] Whitaker R.T., Xue X.W. (2001). Variable-conductance, level-set curvature for image denoising.

